# Mortality Trend for Tumor Correlated Immune System in Hyperendemic Area of HCV Infection in Southern Italy: Joinpoint Analysis

**DOI:** 10.5812/hepatmon.12725

**Published:** 2013-08-27

**Authors:** Maurizio Montella, Matteo Malvezzi, Maria Grimaldi, Flavia Nocerino, Ferdinando Frigeri, Antonio Pinto, Aldo Giudice, Anna Crispo

**Affiliations:** 1 Epidemiology Unit, Istituto Nazionale per lo Studio e la Cura dei Tumori “Fondazione Giovanni Pascale”, IRCCS, Naples, Italy; 2 Department of Epidemiology, “Mario Negri” Farmachological Research Institute, IRCCS, Milan, Italy; 3 Department of Clinical Sciences and Community Health, Università degli Studi di Milan, Milan, Italy; 4 Hematology Unit, Istituto Nazionale per lo Studio e la Cura dei Tumori “Fondazione Giovanni Pascale”, IRCCS, Naples, Italy

**Keywords:** Hepacivirus, Lymphoma, Non-Hodgkin, Mortality, Analysis

## Abstract

**Background:**

In many regions of southern Italy, hepatitis C virus (HCV) infection represents a major health problem (with a prevalence rate between 6% and 13%). HCV is associated with different kinds of neoplasms such as non-Hodgkin lymphomas (NHL), and with auto-immune diseases (cryoglobulinemia), which develop after the virus has caused immune system alterations.

**Objectives:**

To provide updated information on trends in mortality in a major metropolitan area of southern Italy from NHL, multiple myeloma and Hodgkin disease we analyzed cancer mortality data from 1988 to 2009.

**Materials and Methods:**

Mortality data were extracted from National death certificates by age groups, gender, residence and cause of death by the Italian national institute of statistics (ISTAT). Age-standardized mortality rates (SMR) were computed applying the direct method and using the world standard population. To quantify the recent direction of temporal trends in older populations over time, truncated age-adjusted mortality rates were calculated for people aged 65 years and older. Cancer mortality trends were described using their estimated annual percent change (EAPC) and related 95% Confidence Interval (CI).

**Results:**

Statistically significant increasing EAPC was found among women for NHL (+2.0% / year), while statistically significant decrease was found among men and women for HD (-3.5% / year, -3.4% / year, respectively). No statistically significant EAPC was found for multiple myeloma.

**Conclusions:**

The association between viral hepatitis and NHL in the area of interest might provide some degree of explanation to this finding. Our data confirm that due to epidemic infection of HCV in the area of Naples, a high mortality for NHL persists, moreover the adoption of standard therapeutic protocols administered in full accordance with an evidence-based approach and current guidelines explain reduced mortality from Hodgkin lymphomas.

## 1. Background

In many regions of southern Italy, hepatitis C virus (HCV) infection represents a major health problem (with a prevalence rate between 6 and 13%) (1). In the past year the factors that brought about the prevalence of HCV were the extensive use of glass syringes, poor education and promiscuous poverty. Moreover sanitary procedures (surgical and dental interventions) and the health system in general are still less efficient and less meticulous than in Northern Europe and in the rest of Italy (2). Infection with hepatitis B virus (HBV) and hepatitis C virus (HCV) is a major risk factor for HCC in developed countries, but HCV is associated with different kinds of neoplasms such as non-Hodgkin lymphomas (NHL), and with auto-immune diseases (cryoglobulinemia), which develop after the virus has caused immune system alterations (3-6). An association with multiple myelomas has been noted, while no association has been shown for Hodgkin disease (7, 8). HCV is an RNA virus that cannot be integrated with the host genome; it can, however, exert its oncogenetic potential indirectly by contributing to the modulator effects of the host immune system, probably through a capacity to elude the immune system (9).

## 2. Objectives

To provide updated information on trends in mortality in a major metropolitan area of southern Italy (Naples 3,500,000 inhabitants) from non-Hodgkin lymphoma, multiple myeloma and Hodgkin disease we analyzed cancer mortality data for all ages and for 65+ truncated age group from 1988 to 2009.

## 3. Materials and Methods

Mortality data were extracted from National death certificates by age (5 years) groups, gender, residence and cause of death. These records were made available by the Italian national institute of statistics (ISTAT). The time window considered spanned from 1988 to 2009. International classification of diseases (ICD-9 and ICD-10) changed twice from 1998 to 2008 therefore records related to cancer deaths were re-coded according to the tenth revision of the ICD.

### 3.1. Data Analysis

From the matrices of certified deaths and resident population we extracted mortality data related to 5-year age-groups for every calendar year between 1988 and 2009. Data for the years 2004 and 2005 were not available.

### 3.2. Statistical Analysis

Age-standardized mortality rates (SMR) were computed for each 5-year age group, by gender, primitive cancer site and province applying the direct method and using the world standard population. To quantify the recent direction of temporal trends in older populations over time, truncated age-adjusted mortality rates were calculated for people aged 65 years and older. Cancer mortality trends between 1988 and 2009 were analyzed using joinpoint regression, using the program provided by the United States surveillance epidemiology and end results (US SEER) (10). Cancer mortality trends were described using their estimated annual percent change (EAPC) and related 95% confidence interval (CI).

## 4. Results

For all ages between 1988 and 2009, in men from Naples, SMR for non-Hodgkin lymphoma increased from 3.6 to 4.2 per 100,000 among women, SMRs increased slightly from 2.0 to 2.4 per 100.000. SMRs for multiple myeloma, in the study period, stay the same in both sexes (from 1.1 to 1.7 per 100,000 and from 1.6 and 1.5 per 100,000 in men and women respectively). Whereas for Hodgkin disease during the period 1988 to 2009 decreased SMRs were observed either for men or women (from 0.9 to 0.4 per 100,000 and 0.8 to 0.4 per 100,000 respectively). In the 65+ truncated age group from Naples, SMRs for non-Hodgkin lymphoma increased from 20.0 to 26.6 per 100.000 in men and from 8.7 to 16.0 per 100.000 in women. Similarly to the results of all ages in the elderly for multiple myeloma SMRs were uniform during the study period in both sexes (from 11.2 to 16.6 per 100,000 and 15.3 to 15.8 per 100,000 in men and women respectively). For Hodgkin disease, decrease SMRs were observed for men and women (2.8 to 2.0 per 100.000 and from 3.4 to 1.5 per 100.000, respectively).

**Figure 1.**
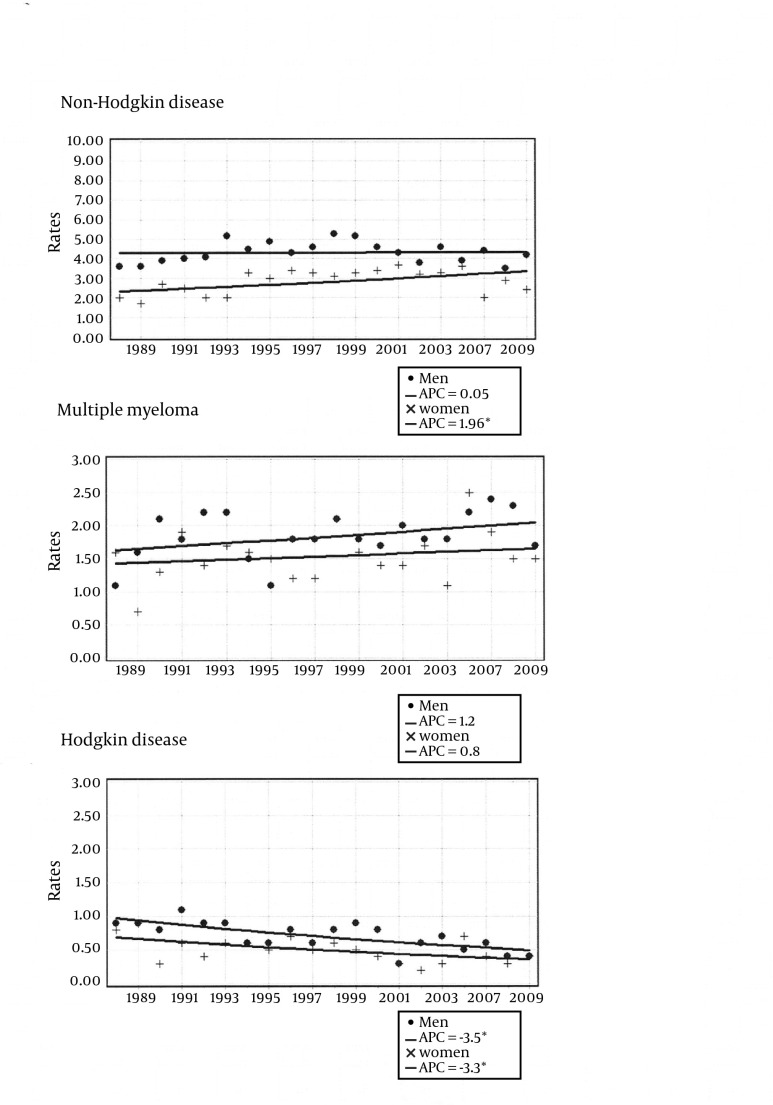
All Ages Joinpoint Analysis for NHL, Multiple Myeloma, Hodgkin’s Disease in the Metropolitan Area of Naples According to Gender, From 1988 to 2009 Men data points (circle), men joinpoint model (solid line). Women data points (cross), women joinpoint model (solid line)

**Figure 2.**
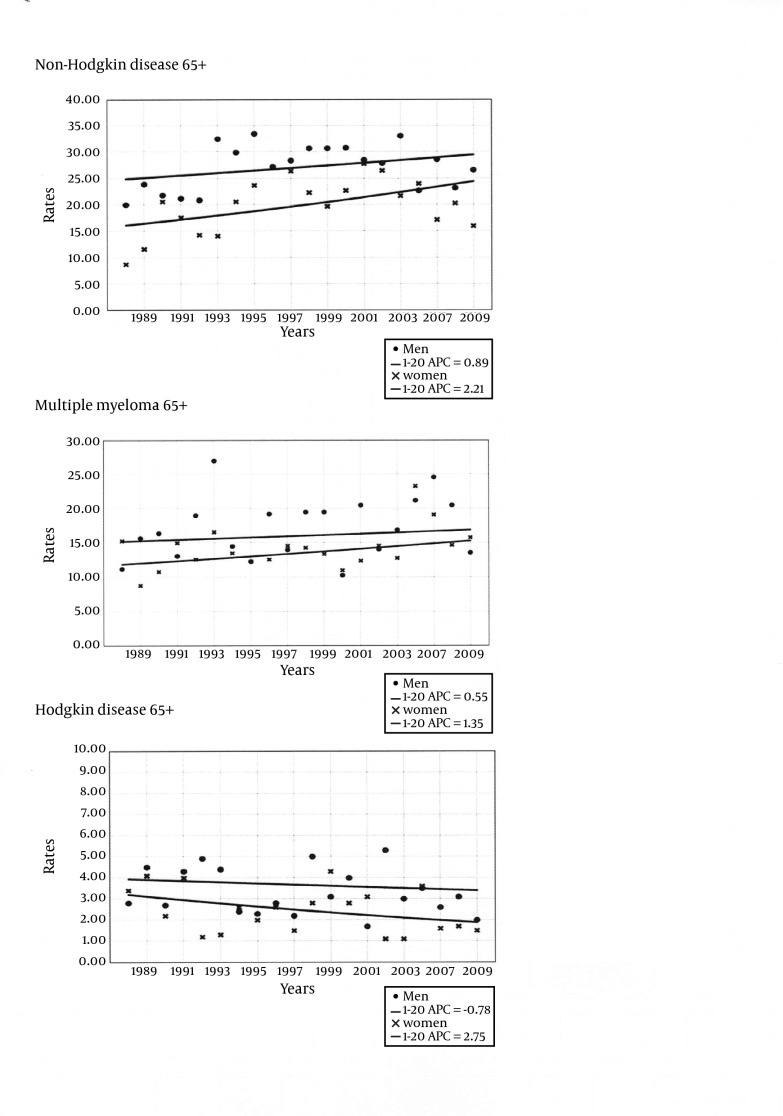
65+ Truncated Age-Group Joinpoint Analysis of Age-Standardized Death Rates for NHL, Multiple Myeloma, Hodgkin’s Disease in the Metropolitan Area of Naples According to Gender, From 1988 to 2009 Men data points (circle), men joinpoint model (solid line). Women data points (cross), women joinpoint model (solid line)

Results from the joinpoint regression analysis are shown in Figure 1 for all ages and in Figure 2 for the elderly (65 over); EAPC values are given by sex for non-Hodgkin lymphoma, multiple myeloma and Hodgkin disease from 1998 to 2009.

Statistically significant increasing EAPC was found among women for NHL (+2.0% / year), while statistically significant decrease was found among men and women for HD (-3.5% / year, -3.4% / year, respectively). No statistically significant EAPC ware found for multiple myeloma. The 65+ truncate age group had no significant NHL increasing EAPC both in men (EAPC = +0.01% / year, EAPC = +0.89% / year; respectively), and in women (EAPC = +1.96% / year, EAPC = +2.21% / year; respectively). For multiple myeloma the rise was only observed in women for all ages, finally for Hodgkin disease the negative EAPC values were smaller in the elderly compared to the all ages group both in men (EAPC = -3.5% / year, EAPC = -0.78% / year; respectively) and in women (EAPC = -3.4% / year, EAPC = -2.75% / year; respectively).

## 5. Discussion

Our results for non-Hodgkin lymphoma are not consistent with the decrease in mortality seen in the Italian data (APC = -3.1% / year in men and APC = -3.9% / year in women; APC = -1.4% / year in men and APC = -1.7% / year in women over 65 years) (11). We observed significantly increasing cancer mortality trends from non-Hodgkin lymphoma in women from the metropolitan area of Naples, which were somewhat consistent with the non-significant increases reported for females in elderly women aged 65 and older. The available evidence on the association between viral hepatitis and non-Hodgkin lymphoma in the metropolitan area of interest might provide some degree of explanation to this finding. Indeed, in a previous hospital-based case-control study including participants from Naples, HCV infection was associated with a significantly increased risk of non-Hodgkin lymphoma, with the fraction of cases attributable to HCV being 12.4% (7) These results were consistent with those from a previous study including people from Campania and assessing the association of HCV with a number of tumors correlated with the immune system. HCV infection was associated with greater risk not only for B-cell non-Hodgkin lymphoma, but also for multiple myeloma (odds ratios (OR) 3.7 and 95% CI 1.9-7.4 and OR 4.5, and 95% CI 1.9 – 10.7, respectively) (8, 12).

A possible explanation for this association could be the ability of HCV to infect early differentiation stages of hematopoietic cells. In this case, a subsequent mutational event, occurring before complete maturation, (e.g. before germinal center entry) gives an aggressive disease (13). The mechanisms responsible for persistence of viral infection and for the cellular lesion are not well understood. Despite an active response from the host, HCV has the capacity to elude the immune system. It is believed that the quasispecies nature of HCV is one of the major mechanisms allowing the virus to develop chronic infection (6, 9).

Existing literature suggest that exposure to polychlorinatedbiphenyls (PCBs) contributes to NHL risk. In a recent study showed that polychlorinated biphenyls (PCBs) exposure can compromise the immune surveillance mechanism. Highly chlorinated PCNs with strong affinity for hydrocarbon receptor (Ahr) are potent immonotoxicants that increase NHL risk. All PCBs can induce reactive oxygen species formation, genotoxic effects, immune suppression and inflammatory response to various extents and through different pathways (14). Nevertheless the high prevalence of HCV in the Neapolitan area, may largely contribute to explain the higher mortality rate for NHL in our study (1, 7, 8).

Our data confirm that due to epidemic infection of HCV in the area of Naples, a high mortality for NHL persists, moreover the adoption of standard therapeutic protocols and integrated treatments administered in full accordance with an evidence-based approach and current guidelines explain reduced mortality from Hodgkin lymphomas, especially in patients younger than 65 years of age.
